# The rise in antimicrobial resistance: An obscure issue in COVID-19 treatment

**DOI:** 10.1371/journal.pgph.0000641

**Published:** 2022-07-13

**Authors:** Yogendra Shrestha, Ravi Kurikempannadoddi Shivalingegowda, Melkote Jyotiprakash Avinash, Sharath Babu Hagalahalli Kenchegowda, Jeet Bahadur Moktan, Sreenivas Murthy Doddasamiah, Ramesh Mahadev Tambat, Deepanjali Girish Golshetty, Vakkalagadda Siva Ganesh, Rajesh Venkataraman

**Affiliations:** 1 Department of Pharmacy Practice, Sri Adichunchanagiri College of Pharmacy, Adichunchanagiri University, B. G. Nagara, India; 2 Department of Otorhinolaryngology and Head & Neck Surgery, Adichunchanagiri Institute of Medical Sciences, Adichunchanagiri University, B. G. Nagara, India; 3 Department of General Surgery, Sapthagiri Institute of Medical Sciences and Research Centre, Bangalore, India; 4 Department of Orthopaedics, Jayanagar General Hospital, Bangalore, India; 5 Department of Paediatrics, Adichunchanagiri Institute of Medical Sciences, Adichunchanagiri University, B. G. Nagara, India; 6 Department of Pharmacology, Sri Adichunchanagiri College of Pharmacy, Adichunchanagiri University, B. G. Nagara, India; South African Medical Research Council, SOUTH AFRICA

## Abstract

A saturated health care system with a lack of evidence-based antiviral medicine and ignorance of antimicrobial stewardship during pandemics has prompted clinicians to prescribe a broad-spectrum antibiotic more often. A prospective, cross-sectional study of COVID-infected patients was conducted to gain insight into antibiotic prescribing practices and their impact on antimicrobial resistance. The antibiotic susceptibility test was performed using the disc diffusion method. 318 patients met the study’s inclusion criteria, with a mean age of 46 years and 55% (175) of them being males. Antibiotics were prescribed for 93.72% (209) of mild cases, 92.45% (49) of moderate cases, 96.15% (25) of severe cases, and 100% (16) of critical cases of COVID-19. A total of 95 samples were sent in for culture and antibiotic sensitivity testing, with 58.95% (56) confirming growth. The majority of the growth was found to contain E. coli (14). In 54.9% of cases, antibiotics with less than 50% sensitivity to curing bacterial infection were detected. In the study, we found that antibiotics were being used unnecessarily in excessive quantities and that more than half of the antibiotics were less sensitive to isolated bacteria.

## Introduction

Antibiotics are being utilized unnecessarily in the treatment of COVID-19. Excessive and needless antibiotic use raises the risk of antimicrobial resistance (AMR) in the future by promoting the emergence of multidrug-resistant (MDR) bacteria [1]. The World Health Organization (WHO) recently revised its guidelines against the use of empirical antibiotics in COVID-19 [2], which has sparked concerns about AMR. Unless there is a clinical sign of a bacterial infection, recent guidance discourages antibiotic therapy or prophylaxis for individuals with mild COVID-19 symptoms or those with suspected or confirmed moderate COVID-19 illness [3]. Antibiotic resistance patterns must be transparently monitored and reported to combat bacterial resistance [4].

COVID-19 resembles bacterial pneumonia in conjunction with clinical presentation, including fever, cough, and lung infiltrates [5]. It could be a reason to treat ill patients with empirical antibiotics as a preventive measure. On the other hand, a saturated health care system with a lack of evidence-based antiviral medicine and ignorance of antimicrobial stewardship during pandemics has prompted clinicians to prescribe broad-spectrum antibiotics more often [2, 6]. Zhou F et al. observed that antibiotics were given to 95% of the patients, antivirals were given to 21%, and 15% of them had suffered from secondary bacterial infection [7]. Goyal P et al. observed that only 5.6% of patients experienced bacteraemia [8].

Here, the study intends to get insight into antibiotic prescribing practices in COVID-19 treatment and its impact on antibiotic resistance.

## The method and methodology

### Study design

A prospective, cross-sectional study was conducted in the COVID ward of Adichunchanagiri Hospital & Research Centre (AH & RC) after obtaining approval from the Intuitional Ethical Committee (IEC/AH & RC/AC/002/2021).

### Study participants

The study included all patients diagnosed using the diagnostic method reverse transcription- polymerase chain reaction (RT-PCR) and admitted to the COVID wards between August 1st and November 30th, 2021, regardless of gender. The study did not include those diagnosed with other diagnostic methods than RT-PCR.

### Study procedures and data collection

All COVID-19 patients hospitalized in AH & RC’s COVID wards were assessed for inclusion criteria, and those who met them were considered for the study. Although the study was part of a daily medication evaluation by a clinical pharmacist for the patient’s safety, informed consent was obtained from patients who were capable of giving it or from the caretaker for severe and critical patients.

The data collection form was designed and validated, contains various sections which include demographic details (age, gender, comorbidities, and Coronavirus prevention measures), the severity of the disease (*mild*, *moderate*, *severe*, *and critical*) [9], anti-microbial medications prescribed (antiviral, antibiotic, and antiprotozoal), and an antibiotic susceptibility test (AST) report. Relevant information was gleaned from the patient’s case report. During data collection, patients and their caretakers were briefed about coronavirus prevention strategies and asked if they were following them appropriately. The sample processing for AST was not enforced on the treating physician; instead, it was left to their discretion.

### Antibiotic susceptibility test

AST was performed by the clinical microbiologist at the central laboratory of AH&RC as a part of routine diagnostics. In brief, samples were inoculated into MacConkey Agar, Blood Agar, and Chocolate Agar and incubated for 24 hours before AST. The disc-diffusion method is used on Muller-Hinton agar for AST. The Clinical and Laboratory Standards Institute (CLSI) guidelines 2020 were used to interpret the result of the AST test [10].

### Data analysis

All of the collected data was imported into MS Excel, and statistical analysis was performed employing the Statistical Package for the Social Sciences (SPSS) version 26 program. The frequency and percentage of all nominal variables such as age, gender, coronavirus prevention measures, the severity of the disease, bacterial isolation, antibiotic use, and antibiotic resistance were measured. The Chi-Square test was used to find out the relationship between the different variables. A significant association was defined as one with a P-value less than 0.05.

## Result

During the study’s duration, 356 Covid-infected individuals were admitted to the hospital, with 38 of them failing to satisfy the study’s inclusion criteria, bringing the number of subjects to 318. There were 55% (175) males and 45% (143) females. The average age was 45.33 ± 17.33 with an interquartile range (IQR) of 26.25 (Q3 = 57.25 and Q1 = 31). The majority of patients were above the age of 60, representing 19.8% (63) of the total, while the minority of patients were under the age of 20, representing only 4.7% (15) of the total. At least one comorbidity was present in 25.5% (81) of the patients. During the study period, 5.7% (18) of the patients lost their lives. Overall, 87.7% (279) of patients said they used hand sanitizer, 79.9% (254) said they cleaned their hands with soap and water, 58.5% (186) said they wore a mask, and 51.3% (163) said they maintained a safe distance. COVID-19 symptoms were classified as mild in 70.13% (223) of the patients, moderate in 16.67% (53) of the patients, severe in 8.2% (26) of the patients, and critical in 5.0% (16) of the patients (Table 1).

**Table 1:** Distribution of patient’s characteristics, antimicrobial prescribed, and the severity of the disease.

Patient’s characteristic			Antibiotic Prescribed		Total number	Percentage (n = 318)	
			Yes	No			P-value
Gender (Total participant = 318)	Male		165	10	175	55%	0.163
	Female		132	11	143	45%	
Severity	Mild		207 (93%)	14	223	70.13%	0.644
	Moderate		50 (94.3%)	3	53	16.67%	
	Severe		25 (96.15%)	1	26	8.2%	
	Critical		16 (100%)	0	16	5%	
Comorbidity					80	25.2%	
Death					18	5.7%	
Agreed to use Hand Sanitizer					279	87.7%	
Agreed to Wash Hands					254	79.9%	
Agreed to Using Mask					186	58.5%	
Agreed to maintain safe distance					163	51.3%	
Antibiotic prescription		Total			299	94%	
		Single			201	63.2%	
		Two			78	24.5%	
		Three			18	5.7%	
		Four			2	0.6%	
Antiprotozoal prescription					252	79.3%	
Antiviral prescription					245	77%	
Sent for Culture					95	29.87%	
Growth					56 (58.95%)	17.6%	

**Fig 1:**
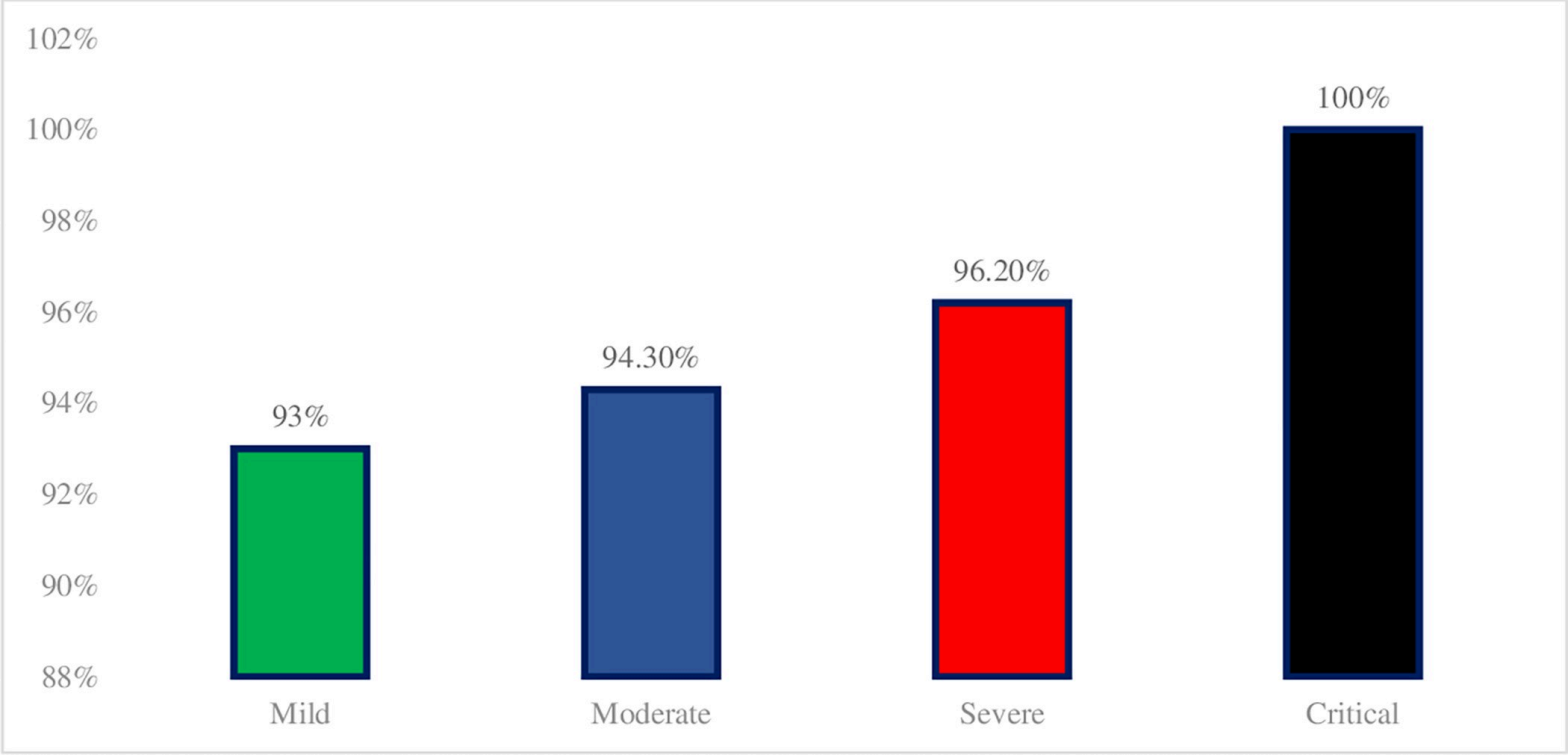
Distribution of antibiotics prescribed for the treatment of COVID-19 based on severity.

Antibiotics were prescribed for 93.7% (298) of the prescriptions, antiprotozoal (HCQS & Ivermectin) were prescribed for 79.3% (252), and antiviral (Oseltamivir, Favipiravir, Remdesivir, and Ritonavir/Lopinavir) were prescribed for 77% (245). Antibiotics were prescribed for 93% (207) of mild cases, 94.3% (50) of moderate cases, 96.15% (25) of severe cases, and 100% (16) of critical cases of COVID-19 (Table 1 & Fig 1). Azithromycin was prescribed at 73.6% (234), followed by piperacillin/tazobactam at 14.5% (46), ceftriaxone 13.5% (43), meropenem 6.9% (22), doxycycline 6.6% (21), amoxicillin/clavulanic acid 3.5% (11), ofloxacin 3.1% (10), amikacin 2.8% (9), metronidazole 2.5% (8), colistin 1.3% (4), ciprofloxacin 0.9% (3), cefotaxime 0.9% (3), cefixime 0.6% (2), linezolid 0.6% (2), and rifaximin 0.6% (2). 63.2% (201) were prescribed single antibiotics, 24.5% (78) with two antibiotics in a prescription, 5.7% (18) with triple antibiotics in a prescription, and 0.6% (2) with quad antibiotics in a prescription (Tables 1 & 2). Combinations of azithromycin and ceftriaxone were predominant, followed by azithromycin and piperacillin/tazobactam, azithromycin and meropenem, and azithromycin and amikacin.

**Table 2:** Distribution of antibiotics prescribed.

Antibiotic	Total number	Percentage
Azithromycin	235	73.9%
Piperacillin/tazobactam	46	14.5%
Ceftriaxone	43	13.5%
Doxycycline	21	6.6%
Amikacin	9	2.8%
Metronidazole	8	2.5%
Meropenem	22	6.9%
Ciprofloxacin	3	0.9%
Cefixime	2	0.6%
Cefotaxime	3	0.9%
Amoxicillin/clavulanic	11	3.5%
Ofloxacin	10	3.1%
Rifaximin	2	0.6%
Linezolid	2	0.6%
Colistin	4	1.3%

**Fig 2:**
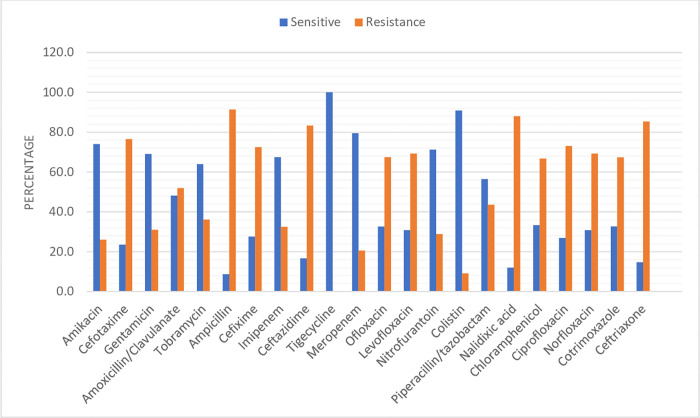
The distribution of antibiotic sensitivity and resistance was based on the results of the AST test.

95 (29.9%) specimens were sent for AST testing out of 318 patients, with 16.59% (37) being mild, 66% (35) being moderate, 80.8% (21) being severe, and 12.5% (2) being critical. 58.9% (56) of them confirmed growth. *E*. *Coli* was isolated in the majority of the samples, 25% (14) followed by *Klebsiella Pneumonia* 17.9% (10), *Pseudomonas spp*. 14.3% (8), *Enterobacter spp*. 10.7% (6), *Enterococcus* 10.7% (6), *Staphylococcus* [MR CONS] 8.9% (5), *non-fermenting gram-negative bacteria* [NFGNB] 7.1% (4), *Providencia Stuartii* 1.8% (1), *Proteus Mirabillis 1*.*8*% (1), and *Citrobacter Freundii* 1.8% (1) (Table 3). Regardless to the isolated bacteria, the overall AST result was 100% sensitive to tigecycline, 90.9% to colistin, 79.9% to meropenem, 74% to amikacin, 71.2% to nitrofurantoin, 69% to gentamicin, 67.5% to imipenem, 63.9% to tobramycin, 56.4% to piperacillin/tazobactam, 48.1% to amoxicillin/clavulanate, 33.3% to chloramphenicol, 32.7% to cotrimoxazole, 32.6% to ofloxacin, 30.8% to norfloxacin, 30.8% to levofloxacin, 27.5% to cefixime, 26.9% to ciprofloxacin, 23.5% to cefotaxime, 16.7% to ceftazidime, 14.6% to ceftriaxone, 12% to nalidixic acid, and 8.7% to ampicillin (Table 4 & Fig 2).

**Table 3:** Distribution of isolated bacteria.

Organisms Detected	Frequency	Percentage (n = 56)
*CITROBACTER FREUNDII*	1	1.8%
*E*. *COLI*	14	25%
*ENTEROBACTER SPP*	6	10.7%
*KLEBSIELLA PNEUMONIA*	10	17.9%
*NFGNB*	4	7.1%
*PROTEUS MIRABILLIS*	1	1.8%
*PROVIDENCIA STUARTII*	1	1.8%
*PSEUDOMONAS SPP*	8	14.3%
*STAPHYLOCOCCUS [MR CONS]*	5	8.9%
*ENTEROCOCCUS*	6	10.7%
TOTAL	56	100%

**Table 4:** Distribution of antibiotics sensitive and resistant irrespective of organism detected.

Name	Total Isolated Organism (n = 56)			Excluding Not Tested	
	Sensitive	Resistance	Not tested	Sensitive	Resistance
Amikacin	66.1% (37)	23.2% (13)	10.7% (6)	74%	26%
Cefotaxime	21.4% (12)	69.6% (39)	8.9% (5)	23.5%	76.5%
Gentamicin	51.8% (29)	23.2% (13)	25% (14)	69%	31.0%
Amoxicillin/Clavulanate	46.4% (26)	50% (28)	3.6% (2)	48.1%	51.9%
Tobramycin	41.1% (23)	23.2% (13)	35.7% (20)	63.9%	36.1%
Ampicillin	7.1% (4)	75% (42)	17.9% (10)	8.7%	91.3%
Cefixime	19.6% (11)	51% (29)	28.6% (16)	27.5%	72.5%
Imipenem	48.2% (27)	23.2% (13)	28.6% (16)	67.5%	32.5%
Ceftazidime	12.5% (7)	62.5% (35)	25% (14)	16.7%	83.3%
Tigecycline	16.1% (9)	0	83.9% (47)	100%	0
Meropenem	55.4% (31)	14.3% (8)	30.4% (17)	79.5%	20.5%
Ofloxacin	25% (14)	51.8% (29)	23.2% (13)	32.6%	67.4%
Levofloxacin	28.6% (16)	64.3% (36)	7.1% (4)	30.8%	69.2%
Nitrofurantoin	66.1% (37)	26.8% (15)	4.1% (4)	71.2%	22.8%
Colistin	17.9% (10)	1.8% (1)	80.4% (45)	90.9%	9.1%
Piperacillin/tazobactam	39.3% (22)	30.4% (17)	30.4% (17)	56.4%	43.6%
Nalidixic acid	10.7% (6)	78.6% (44)	10.7% (6)	12%	88%
Chloramphenicol	3.6% (2)	7.1% (4)	89.3% (50)	33.3%	66.7%
Ciprofloxacin	25% (14)	67.9% (38)	7.1% (4)	26.9%	73.1%
Norfloxacin	28.6% (16)	64.3% (36)	7.1% (4)	30.8%	69.2%
Cotrimoxazole	28.6% (16)	58.9% (33)	12.5% (7)	32.7%	67.3%
Ceftriaxone	10.7% (6)	62.5% (35)	26.8% (15)	14.6%	85.4%

There were no statistically significant differences in the antibiotics prescribed between the severity of COVID-19 and the p-value obtained was 0.644. Similarly, the antibiotics prescribed had a p-value of 0.163, indicating that no statistically significant differences existed between the genders. However, statistically significant differences in the severity of diseases were observed in the sample collection for the antibiotic susceptibility test (AST), with a p-value of 0.000.

## Discussion

With this study, we intended to get insight into antibiotic prescribing practices in COVID-19 treatment and its impact on antibiotic resistance. In the study, we observed that the antibiotic prescribed was unaffected by the severity of COVID-19. At least 93.7% of the patients were prescribed at least one antibiotic, and azithromycin alone accounted for 73.6%. Similar findings were reported by WHO and Getahun H et al. [3, 11]. Antibacterial medications were preferred over antiviral drugs, which is similar to the results of Zhou F et al [7]. However, the number of antivirals used was seen to have increased. Antibiotics were prescribed in 93% of mild cases and 94.3% of moderate cases, despite COVID-19’s revised guidelines prohibiting the use of empirical antibiotics. It could be influenced by the findings of studies aimed at enhancing treatment outcomes by combining antibiotics with antiviral or antiprotozoal drugs when no cure is available [12–15].

Although antibiotics were started before the specimens were collected for AST, the growth was present in 58.9% of the total specimens sent for AST. This shows that antibiotics are being used inappropriately and the process of specimen collection was contrary to the Treatment Guidelines for Antimicrobial Use in Common Syndromes [16]. We found that *E*. *coli*, *K*. *Pneumoniae*, *and Pseudomonas spp*. were the most commonly isolated bacteria, while Marina G et al. [17] reported that *Pseudomonas aeruginosa* and *Enterobacterales* were the most prevalent in their study. The isolation of organisms is being influenced by the geographical region. 54.5% (12) of the antibiotics used for AST showed more than 50% resistance to the isolated pathogens. Antibiotics such as ampicillin, nalidixic acid, ceftriaxone, ceftazidime, cefotaxime, cefixime, and ciprofloxacin, which are commonly prescribed, were resistant in more than 70% of AST results. The inappropriate and overuse of antibiotics without an indication could be a cause of the issue. It’s also possible that the current pandemic’s focus on minimizing the immediate impact on individuals has obscured the longer-term threat of AMR [6].

Hand sanitizer was used by 92.43% of patients, indicating that it is an important component of COVID-19 transmission prevention awareness campaigns. Ochwoto M et al. reported that half of the hand sanitizers tested have efficacy levels that are lower than the WHO formulation in their study [18]. The pandemic’s desperate circumstances prevented quality assurance of the supplied hand sanitizer. Ineffective hand sanitizers encourage the growth of drug-resistant bacteria, increasing the risk of antibiotic cross-resistance [19].

The findings of the investigation highlight the terrible situation of antibiotics. Its inappropriate and overused nature can be more fatal if a pathogen develops multi-drug resistance. To save antibiotics in the future, no compromises should be made in the implementation of antimicrobial stewardship programs. AMR is caused by a variety of factors, including antibiotic overuse and misuse, as well as exposure to low-quality biocidal agents.

Our study’s limitation is that it was a single-center study with a small sample size, and we assessed antibiotic resistance independently of the isolated bacterium. The fact that AH&RC is located in a rural part of the Mandya district may have impacted its decision to ignore coronavirus protective measures such as safe distance and mask use. Further multi-center studies with a large sample size are required to demonstrate the impact of the COVID pandemic on AMR.

## Conclusion

In the study, we identified that antibiotics were being used unnecessarily in excessive amounts and that more than half of the antibiotics tested were less sensitive to curing bacterial illness, regardless of the isolated bacterium. The current pandemic’s focus on reducing the immediate impact on humans may have hidden the longer-term threat of AMR, overuse of antibiotics, and exposure to inadequate biocidal agents, which might lead to an increase in AMR. To corroborate the findings, a multi-center study with larger sample size is necessary.
